# Alcohol dehydrogenase 1B (*ADH1B*) genotype, alcohol consumption and breast cancer risk by age 50 years in a German case–control study

**DOI:** 10.1038/sj.bjc.6602608

**Published:** 2005-05-10

**Authors:** C Lilla, T Koehler, S Kropp, S Wang-Gohrke, J Chang-Claude

**Affiliations:** 1 Division of Clinical Epidemiology, Deutsches Krebsforschungszentrum, Im Neuenheimer Feld 280, Heidelberg 69120, Germany; 2 Molecular Biology Laboratory, Department of Obstetrics and Gynecology, University of Ulm, Frauensteige 14, Ulm 89075, Germany

**Keywords:** alcohol dehydrogenase, polymorphism, gene–environment interaction, breast cancer

## Abstract

In a population-based study of 613 cases and 1082 controls, alcohol dehydrogenase 1B (*ADH1B*) genotype was not an independent risk factor for breast cancer, athough the possibility was raised that it modifies risk associated with high levels of alcohol consumption (OR 1.1, 95% confidence interval (CI) 0.8–1.6 for ADH1B*1/*1 genotype *vs* 0.2, 95% CI 0.1–1.0 for ADH1B*2 carriers).

Alcohol consumption is one of the few modifiable risk factors for breast cancer (Singletary and Gapstur, 2001; Collaborative Group on Hormonal Factors in Breast Cancer, 2002), and we also observed a dose-dependent effect of alcohol intake on breast cancer risk in a case–control study of women up to age 50 years in Germany (Kropp *et al*, 2001). However, drinking behaviour as well as susceptibility to alcohol-induced carcinogenesis may be influenced by individual genetic make-up. In this context, class I alcohol dehydrogenases (ADHs), which are important enzymes in the major pathway of alcohol metabolism *in vivo* and are expressed in normal mammary epithelium (Triano *et al*, 2003), may be relevant. The alcohol dehydrogenase 1B (*ADH1B*) gene (formerly called *ADH2*) exhibits genetic polymorphisms resulting in altered functional and catalytic properties *in vitro* (Agarwal, 2001). A strongly increased oxidation capability has been associated with the *2 allele (Bosron *et al*, 1983; Eriksson *et al*, 2001), an Arg47His substitution with reported allele frequencies ranging between 0 and 6.8% in Europeans (Brennan *et al*, 2004).

Previously, a significant inverse association between the ADH1B*2 allele and frequency of alcohol consumption was observed in a case-only study (Sturmer *et al*, 2002). We employed a population-based case–control study of women up to age 50 years to examine the potential effect of *ADH1B* genotype on the association between alcohol consumption and breast cancer risk.

## MATERIALS AND METHODS

### Study subjects and data collection

A detailed description of the underlying case–control study has been provided elsewhere (Chang-Claude *et al*, 2000). Patients up to age 50 years with a diagnosis of primary incident *in situ* or invasive breast cancer were recruited between 1 January 1992 and 31 December 1995. Controls were selected randomly from lists of residents supplied by population registries; for each patient, two controls were matched according to exact age and study region. Written informed consent from all participants was obtained. The study was approved by the ethics committee of the University of Heidelberg.

In total, 706 (70.2%) women of the 1005 breast cancer patients who were alive when identified completed the study questionnaire. Of all 2257 eligible controls, 1381 (61.2%) participated. Detailed information on demographic characteristics and various risk factors was elicited by means of a self-administered questionnaire. Alcohol consumption was assessed for three time periods, that is, 15–20, 20–30 and 30–50 years, and for different types of beverages (beer, wine, aperitifs, liquor and spirits). The detailed method for the calculation of average daily alcohol intake has been described previously (Kropp *et al*, 2001).

Menopausal status was defined as the reported state half a year before the reference date, which was the date of diagnosis for cases and the date of completion of the questionnaire for controls. The status of women with previous hysterectomy not accompanied by bilateral oophorectomy was classified as unknown.

The present analysis was restricted to 613 cases and 1082 controls with at least one parent of German nationality and who could be successfully genotyped (three and five failures, respectively).

### Genotyping *ADH1B* by TaqMan PCR

Genomic DNA was extracted from peripheral blood using the QIAamp DNA Blood Kit (Qiagen, Hilden, Germany). Primer and hybridisation probes were designed with Primer Express. Sense primer 5′-CTCTTTATTCTGTAGATGGTGGCTGTAG-3′ and antisense primer 5′-GGGTCACCAGGTTGCCACTA-3′ were used to amplify a 76 bp fragment containing the G47A polymorphism of the *ADH1B* gene. Two minor-groove-binding (MGB) DNA probes were synthesised. The probe corresponding to the wild type (5′-FAM-TCTGTCGCACAGATG-MGB-3′) was labelled with 6-FAM, and the probe corresponding to the mutation (5′-VIC-AATCTGTCACACAGATGA-MGB-3′) was labelled with VIC at the 5′-end. The genotypes were analysed in software ‘Sequence Detector’ version 1.7 by procedure allelic discrimination after PCR.

Amplification was performed in a final volume of 25 *μ*l containing 40 ng of DNA, 300 nM of each primer, 200 nM of each probe and 12.5 *μ*l of TaqMan Universal PCR Master Mix (Perkin-Elmer, Weiterstadt, Germany).

In every assay, negative controls as well as controls for the wild type, mutant type and heterozygote were included. The PCR conditions were as follows: 2 min at 50°C plus 95°C for 10 min, followed by 40 cycles of denaturation at 92°C for 15 s, annealing and extension in one step at 60°C for 1 min. Genotyping was performed blinded to case–control status of the blood sample providers.

### Statistical analysis

*χ*^2^ tests were used to assess deviation from Hardy–Weinberg equilibrium. Multivariate conditional logistic regression analysis with 5-year age strata was carried out using the PHREG procedure of the statistical software package SAS release 8.2 (SAS Institute, Cary, NC, USA).

In the multivariate model, we included several relevant variables influencing breast cancer risk (see Table 2). Variables that did not alter the estimates substantially, such as study region, body mass index, use of oral contraceptives and age at menarche, were not included in the analyses presented. Statistical interaction between *ADH1B* genotype and alcohol consumption was tested by using multiplicative interaction terms and evaluated by the likelihood ratio test.

## RESULTS

Selected characteristics of the study population are depicted in Table 1. In all, 65 breast cancer patients (10.6%) and 109 controls (10.1%) were heterozygous or homozygous carriers of the ADH1B*2 allele, corresponding to allele frequencies of 0.06 and 0.05, respectively. The distribution of *ADH1B* genotypes was in Hardy–Weinberg equilibrium (*P*=0.4 for cases and *P*=0.6 for controls).

We did not observe an association between *ADH1B* genotype and several risk factors, including first-degree family history of breast cancer, body mass index, parity, breastfeeding or smoking status (data not shown). However, alcohol consumption was found to differ significantly by *ADH1B* genotype among controls, with a mean average daily alcohol intake of 7.2 g (standard deviation (s.d.) 9.1) in women with ADH1B*1/*1 genotype and 5.8 g (s.d. 7.8) in carriers of the *2 allele (Wilcoxon's rank-sum test *P*=0.01). Results were virtually identical when all study subjects were considered (data not shown).

The analysis revealed no main effect of *ADH1B* genotype on breast cancer risk (adjusted OR for carriers of ADH1B*2 allele vs ADH1B*1/*1 genotype being 1.0, 95% confidence interval (CI) 0.7–1.4). However, multivariate analysis separately for carriers and noncarriers of the ADH1B*2 allele yielded differences in breast cancer risk associated with increasing levels of alcohol intake (Table 2). Among carriers of the *2 allele, there was a significant decreasing trend in breast cancer risk with increasing alcohol consumption (*P*-value for linear trend 0.04), although the odds ratio (OR) was significant only for the category of 12 g or more alcohol per day. In contrast, among women with ADH1B*1/*1 genotype, breast cancer risk increased with increasing alcohol consumption (*P*=0.03). The interaction between *ADH1B* genotype and alcohol intake was borderline statistically significant for the highest category of alcohol consumption (*P*=0.05) but not for low or moderate levels.

## DISCUSSION

Our data raise the possibility of an effect modification of the association between alcohol consumption and breast cancer risk by *ADH1B* genotype in our study population, which was apparent only at the highest consumption category of 12 g or more alcohol per day. We showed previously that breast cancer risk increased significantly with high daily alcohol intake of ⩾19 g in this study population (Kropp *et al*, 2001). Owing to the small number of ADH1B*2 carriers, we were not able to further subdivide the highest alcohol intake category of ⩾12 g day^−1^. Corresponding to a previous case-only study (Sturmer *et al*, 2002), a case-only analysis of our data would yield a statistically significant interaction OR (OR 0.2, 95% CI 0.1–0.6 for ⩾12 g alcohol day^−1^). However, since *ADH1B* genotype and alcohol intake are not independent in our study population, the modifying effect of the *ADH1B* genotype on breast cancer risk associated with alcohol consumption is overestimated in the case-only analysis, partly due to residual confounding by differences in alcohol consumption caused by the genotype (Albert *et al*, 2001). Indeed, indications for an association between *ADH1B* genotype and alcohol consumption, alcoholism or adverse reactions such as flushing were observed in previous studies (Whitfield *et al*, 1998; Borras *et al*, 2000; Loew *et al*, 2003; Neumark *et al*, 2004).

There is still controversy in the literature regarding the effect of *ADH1B* genotype on alcohol pharmacokinetics *in vivo.* Most studies failed to detect differences in blood alcohol or acetaldehyde levels by *ADH1B* genotype (Yamamoto *et al*, 1993; Mizoi *et al*, 1994; Whitfield *et al*, 2001); only one recent study reported a significantly higher alcohol elimination rate in carriers of the ADH1B*2 allele (Neumark *et al*, 2004).

The reduction in breast cancer risk associated with high consumption levels in carriers of the ADH1B*2 allele in our study could therefore be explained by a higher alcohol elimination rate in these subjects. Owing to the low allele frequency of the ADH1B*2 allele in Caucasians, our study had limited power to detect a gene–environment interaction for high alcohol consumption levels and our findings about *ADH1B* genotype as an effect modifier of breast cancer risk associated with high alcohol consumption need confirmation in larger studies.

## Figures and Tables

**Table 1:** Selected characteristics of the study population

	**Cases (*n*=613)**	**%**	**Controls (*n*=1082)**	**%**	***P*-value^a^**
Mean age (s.d.)	42.4 (5.8)		42.6 (5.7)		
					
*Menopausal status*					
Premenopausal	483	78.8	874	80.8	
Postmenopausal	34	5.6	67	6.2	
Unknown	96	15.7	141	13.0	0.30
					
*First-degree family history*					
No	536	87.4	1024	94.6	
Yes	77	12.6	58	5.4	<0.01
					
*Parity*					
0	136	22.2	228	21.1	
1–2 children	414	67.5	680	62.9	
3+ children	63	10.3	174	16.1	<0.01
					
*Breastfeeding* b					
0	153	32.1	247	28.9	
1–12 months	289	60.6	501	58.7	
12+ months	35	7.3	106	12.4	0.01
					
*Education*					
Low	80	13.1	149	13.8	
Intermediate	412	67.2	679	62.8	
High	121	19.7	254	23.5	0.15
					
*Smoking*					
Never active	271	44.2	515	47.6	
Former smoker	132	21.5	258	23.8	
Current smoker	210	34.3	309	28.6	0.05
					
*Alcohol consumption (g day on average^−1^)*					
0	117	19.1	176	16.3	
1–5	233	38.0	454	42.0	
6–11	113	18.4	239	22.1	
12+	150	24.5	213	19.7	0.02
					
*ADH1B genotypes*					
*1/*1	548	89.4	973	89.9	
*1/*2	62	10.1	107	9.9	
*2/*2	3	0.5	2	0.2	0.53^c^

s.d.=standard deviation; *ADH1B*=alcohol dehydrogenase 1B.

a *χ*^2^ test used.

b Among parous women only.

c Fisher's exact test used.

**Table 2:** ORs for breast cancer risk associated with alcohol intake stratified by *ADH1B* genotype

	ADH1B*1/*1 genotype			ADH1B*2 carrier		
	**Cases/controls**	**OR^a^**	**95% CI**	**Cases/controls**	**OR^a^**	**95% CI**
*Alcohol consumption (g day on average^−1^)*						
0	101/147	1 (Ref)		16/29	1 (Ref)	
1–5	200/407	0.74	0.54–1.01	33/47	0.81	0.34–1.91
6–11	104/226	0.70	0.49–0.99	9/13	0.87	0.25–3.02
12+	143/193	1.12	0.78–1.59	7/20	0.29	0.08–1.00
*P*-value for trend^b^		0.03			0.04	

*ADH1B*=alcohol dehydrogenase 1B; OR=odds ratio; CI=confidence interval.

a ORs stratified for age by 5-year intervals; additional adjustment was made for first-degree family history (yes/no), parity (0, 1–2, 3+ children), menopausal status (pre-, postmenopausal, unknown), education (low, intermediate and high) and smoking status (never, former and current active smoking) as categorical variables and breastfeeding (total duration in months) as continuous variable;

b Test for linear trend (mean of categories used).
